# Functional and Proteomic Characterization of *Acanthophis antarcticus* Venom: Evidence of Fibrinogenolytic and Serine Peptidase Inhibitory Activities

**DOI:** 10.3390/toxins17080405

**Published:** 2025-08-13

**Authors:** Monica V. Falla, Enzo P. Sousa, Karen de Morais-Zani, Rodrigo Valladão, Natalia G. Santos, Nathalia C. Galizio, Mariana S. Rodrigues, Heloisa F. Almeida, Adriana R. Lopes, Mauricio N. Moises, Ivo Lebrun, Patrick J. Spencer, Daniel C. Pimenta, Guilherme R. Coelho

**Affiliations:** 1 Laboratório Bioquímica e Biofísica, Instituto Butantan, São Paulo 05503-900, Brazil; m.falla.proppg@proppg.butantan.gov.br (M.V.F.); rodrigo.valladao.esib@esib.butantan.gov.br (R.V.); heloisa.almeida.esib@esib.butantan.gov.br (H.F.A.); adriana.lopes@butantan.gov.br (A.R.L.); m.moyses.proppg@proppg.butantan.gov.br (M.N.M.); ivo.lebrun@butantan.gov.br (I.L.); dcpimenta@butantan.gov.br (D.C.P.); 2 Laboratório de Fisiopatologia, Instituto Butantan, São Paulo 05503-900, Brazil; e.sousa.proppg@proppg.butantan.gov.br (E.P.S.); karen.zani@butantan.gov.br (K.d.M.-Z.); n.galizio.proppg@proppg.butantan.gov.br (N.C.G.); 3 Instituto de Pesquisas Energéticas e Nucleares (IPEN), São Paulo 05508-000, Brazil; mariisoares25@yahoo.com.br (M.S.R.); patrickfrombrazil@gmail.com (P.J.S.); 4 Laboratório de Ecologia e Evolução, São Paulo 05503-900, Brazil

**Keywords:** Elapidae, *Acanthophis*, fibrinogenolytic activity, serine peptidase inhibitors, metallopeptidases, serine peptidases, proteomic

## Abstract

*Acanthophis antarcticus*, commonly known as the death adder, is a venomous Australian snake and a member of the Elapidae family. Due to its robust body and triangular head, it was historically misclassified as a viper. Its venom is known for neurotoxic, hemorrhagic, and hemolytic effects but displays low anticoagulant activity. Although key toxins such as three-finger toxins (3FTxs) and phospholipase A_2_ (PLA_2_) have been previously described, no study has integrated proteomic and functional analyses to date. In this study, we conducted a comprehensive characterization of *A. antarcticus* venom. Reverse-phase high-performance liquid chromatography (RP-HPLC) followed by LC-MS/MS enabled the identification of nine toxin families, with 3FTxs and PLA_2_ as the most abundant. Less abundant but functionally relevant toxins included Kunitz-type inhibitors, CRISP, SVMP, LAAO, NGF, natriuretic peptides, and nucleotidases, the latter being reported here for the first time based on proteomic evidence. Hydrophilic interaction chromatography (HILIC) coupled with MALDI-TOF was used to analyze polar, non-retained venom components, revealing the presence of low-molecular-weight peptides (2–4 kDa). Functional assays confirmed the enzymatic activity of HYAL, PLA_2_, and LAAO and, for the first time, demonstrated inhibitory activity on serine peptidases and fibrinogenolytic activity in the venom of this species. These findings expand our understanding of the biochemical and functional diversity of this venom.

## 1. Introduction

The venom of *Acanthophis antarcticus* has a long history of investigation. In earlier times, this snake was considered a member of the Viperidae family [1,2,3,4,5,6] and was described as the only viperid species on the entire Australian continent [7]. It was only in 1859 that this snake was classified as a member of the *Naja* group within the Elapidae family [8], and this classification was reaffirmed in 1865 [9]. However, it was not until 2000 that *A. antarcticus* was placed alongside *Pseudechis australis* in a phylogenetic tree based on cytochrome b mitochondrial DNA (mtDNA) [10]. Modern approaches have highlighted the need for a taxonomic revision of the genus [11].

This snake is so prominent that it is even mentioned in literary works [12,13]. The first report of *A. antarcticus* toxicity came from Calmette, who observed lethality in guinea pigs with 80 µg of venom [14]. This venom also exhibits neurotoxic, hemorrhagic, and hemolytic properties [3,15]. Ethanol-soluble toxins are responsible for the neurotoxic effects, which resemble curare-like action [15]. Interestingly, this venom lacks significant anticoagulant activity [15,16], and although rhabdomyolysis has been reported in humans [17], this myotoxicity was not observed in experimental models [18]

The Sheumack group discovered the first isolated toxin from *A. antarcticus* venom, Acantophin A, which is a neurotoxic compound [19]. The same group also isolated Alpha-elapitoxin-Aa2d, which blocks the neuromuscular junction postsynaptically [20]. Kim’s group isolated the second toxin from *A. antarcticus* venom, the long-chain neurotoxin Alpha-elapitoxin-Aa2b [21], as well as Short neurotoxin 1, which binds to nicotinic acetylcholine receptors [22]. The research group led by Mirtschin isolated Alpha-elapitoxin-Aa2e, another long-chain postsynaptic neurotoxin [23]. Broady described the phospholipase A_2_ (PLA_2_) isoforms Acanthin 1 and 2 [24], which showed inhibitory effects on platelet aggregation [25]. Anticoagulant effects were further studied by other researchers, first described in patients after envenomation [17]. Additional studies showed that *A. antarcticus* venom is less anticoagulant than venoms from other species of the same genus, such as *A. praelongus* and *A. pyrrhus* [26], but is the most neurotoxic among them [27].

Until 2004, most neurotoxins isolated and characterized from *A. antarcticus* venom acted as postsynaptic toxins [28]. This changed with the discovery of snake presynaptic phospholipase A_2_ neurotoxins (SPANs) in this venom, which revealed the presence of five different short neurotoxins, five long neurotoxins, and six PLA_2_ isoforms [29]. Subsequently, a heterotrimeric PLA_2_ complex of 44.6 kDa was isolated and characterized, showing similarities to taipoxin and acting as a presynaptic neurotoxin [30]. The most recently isolated long alpha-neurotoxin, Alpha-elapitoxin-Aa2a, displays altered affinity for nicotinic receptors due to a leucine-to-arginine substitution in loop II [31].

Mass spectrometry experiments using crude venom revealed the presence of natriuretic peptides (NPs) and Kunitz-type protease inhibitors (KTIs) in venoms of *Acanthophis* species, and phylogenetic comparisons were proposed based on venom composition [32]. Another study using two-dimensional electrophoresis (2D-PAGE) and matrix-assisted laser desorption/ionization time-of-flight mass spectrometry (MALDI-TOF) techniques identified the presence of nerve growth factor (NGF), C-type lectin, snake venom metallopeptidases (SVMPs), L-amino acid oxidase (LAAO), and acetylcholinesterase (AchE) in *A. antarcticus* venom [33]. A transcriptomic analysis of *A. wellsi* venom further expanded the known venom profile of the genus *Acanthophis* by identifying waprin, cysteine-rich secretory proteins (CRISPs), type I and type II alpha-neurotoxins, and a sugar-binding KPG domain in venom lectins [34].

Given the diversity of identified components and the clinical relevance of *A. antarcticus* venom, there is a clear need for a more in-depth characterization of its biochemical and functional composition. The scarcity of reviewed proteomic data for *A. antarcticus* underscores the importance of integrative approaches that combine proteomic and functional analyses. Therefore, the present study proposes a comprehensive investigation of *A. antarcticus* venom using chromatographic, spectrometric, and biochemical techniques, aiming to identify its main toxins and evaluate their enzymatic activities.

## 2. Results and Discussion

### 2.1. Proteome of Acanthophis antarcticus Venom

Proteomic studies on snakes of the *Elapidae* family (1091 reviewed proteins) mainly focus on the Elapinae subfamily, which accounts for 49% of these proteins, with the genus *Naja* standing out (“Elapidae in UniProtKB search (1094)”). In contrast, the *Acanthophiinae* subfamily, to which the genus *Acanthophis* belongs, represents only 24%, with just 11 reviewed proteins attributed to the genus. This scarcity highlights a significant gap in the proteomic knowledge of this group, hindering protein identification using reference databases (Supplementary Material S1—Figures S1 and S2).

The few available studies on *A. antarcticus* venom have mainly focused on the characterization of isolated toxins from the 3FTxs [19,20,21,22,23,29,31] and PLA_2_ families [24,30,35,36], with an emphasis on their neurotoxic effects. Consequently, little is known about the overall venom composition of this species or the functional activity of its proteins. In this context, the present study significantly fills this gap by employing classical biochemical characterization techniques integrated with functional evaluation, combining proteomic and enzymatic approaches in a single analysis. 

**Figure 1:**
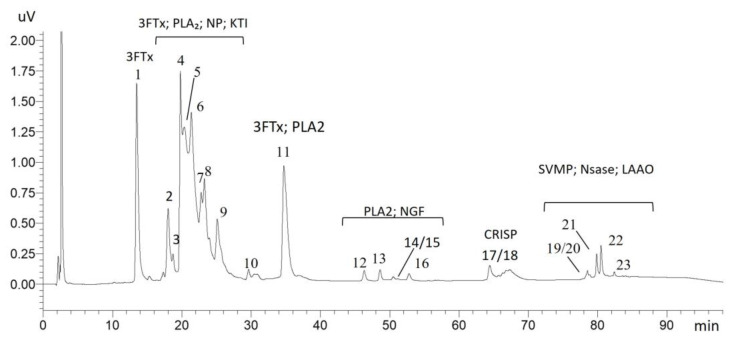
Reverse-phase (RP) chromatogram of *A. antarcticus* venom. The figure shows the numbering of the peaks collected for proteomic analysis, as well as the distribution of the toxin classes identified along the chromatographic profile. Legend: 3FTxs (three-finger toxins), PLA_2_ (phospholipase A_2_), NP (natriuretic peptide), KTI (Kunitz-type inhibitors), NGF (nerve growth factor), CRISP (cysteine-rich secretory protein), SVMP (metalloproteinase), NSase (nucleotidase), and LAAO (L-amino acid oxidase).

The chromatographic profile obtained revealed several peaks distributed over the elution time, indicating a high molecular complexity of the venom (Figure 1). A total of 23 peaks were collected for proteomic analysis, resulting in the identification of toxins belonging to the 3FTxs, PLA_2_, NP, KTI, and NGF families, predominantly within the first 50 min of elution. Between 60 and 90 min, toxins from the CRISP, SVMP, NSase (nucleosidase), and LAAO families were detected. To ensure the reliability of the proteomic data, the following criteria were considered: proteins with a log score >85 and at least one unique peptide. Table 1 provides a more detailed overview of the protein classes identified (Additional information can be found in Supplementary Material S2, Tables S1 and S2).

**Figure 2:**
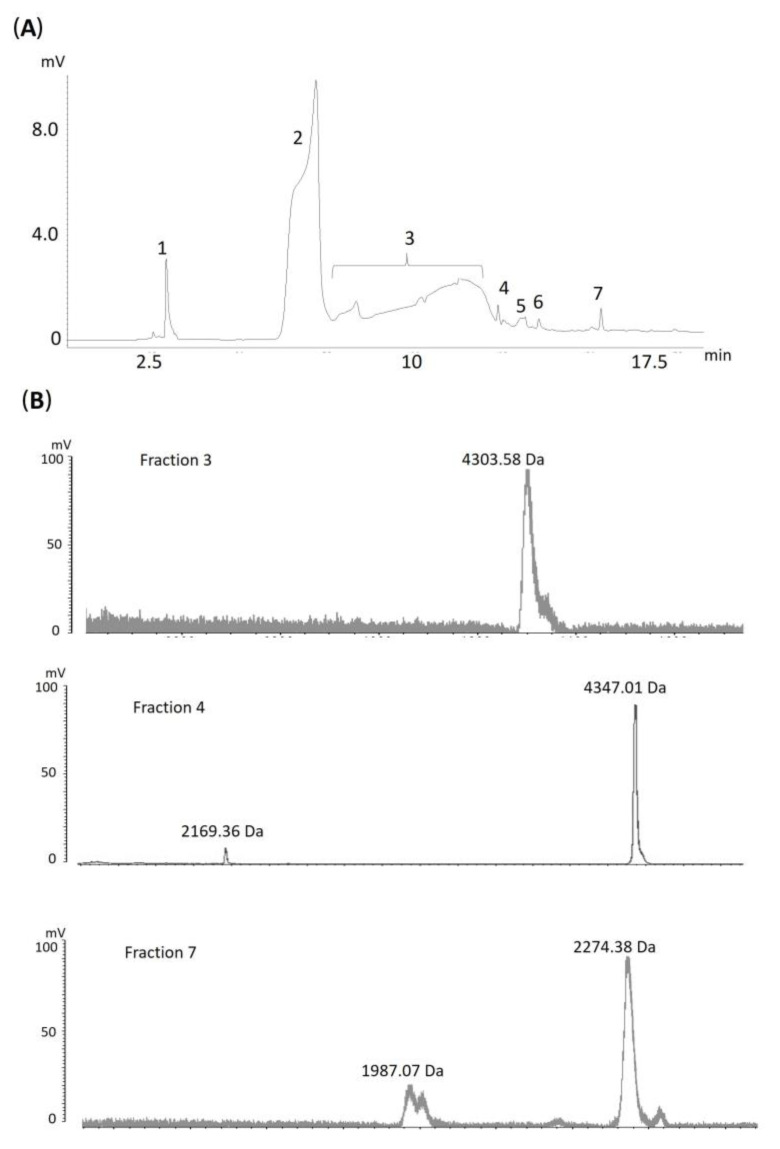
(**A**) Hydrophilic interaction liquid chromatography (HILIC) of *A. antarcticus* venom samples, previously subjected to solid-phase extraction (SPE). (**B**) Mass spectrum obtained by MALDI-TOF from fractions collected by HILIC.

In addition, hydrophilic molecules present in the venom of *A. antarcticus,* which did not interact with the reverse-phase (RP) column, were also investigated. This hydrophilic fraction was collected by solid-phase extraction (SPE) and subsequently separated using hydrophilic interaction liquid chromatography (HILIC). The different peaks obtained were analyzed by MALDI-TOF, resulting in the identification of several molecular masses (Figure 2), which evidences the presence of hydrophilic components that are often overlooked in snake venoms. These findings highlight the informative potential of this fraction, as also demonstrated by Falla et al. (2025), who identified various hydrophilic molecules in venoms from different Viperidae species using hydrophilic molecule separation [37].

Despite the current limited understanding of *A. antarcticus* venom, some studies have identified proteins beyond the 3FTxs and PLA_2_ families. Fry et al. (2002) [32] reported the presence of NP, KTI, 3FTxs, and PLA_2_ toxins in different species of the genus *Acanthophis*. However, in *A. antarcticus* specifically, PLA_2_ and NP were not detected, which contrasts with the present study, where both toxin types were identified in this species [32].

Birrell (2007), using two-dimensional electrophoresis (2D-PAGE) followed by proteomic analysis, identified toxins from the C-type lectin-like, 3FTxs, NGF, LAAO, SVMP, KTI, PLA_2_, and AChE families in *A. antarcticus* venom [33]. These findings are consistent with the data obtained in our analyses, except for C-type lectin-like and AChE toxins, which were not detected in our proteomic analysis under the adopted criteria.

Jackson et al. (2013) conducted a transcriptomic analysis of the venom gland of *A. wellsi*, in which transcripts encoding CRISP, KTI, lectins, NP, PLA_2_, SVMP, 3FTxs, and waprin were identified [34]. Although *A. wellsi* is a different species, it belongs to the same genus. Given the lack of transcriptomic data for *A. antarcticus*, this information was used as a comparative basis for the proteomic data obtained in the present study Here, toxins showing high similarity to members of the 3FTx, PLA_2_, KTI, and SVMP families were identified at the protein level in the venom of *A. antarcticus*, demonstrating the conservation of these toxin families within the genus *Acanthophis*.

#### 2.1.1. Main Toxin Classes Identified

**Figure 3:**
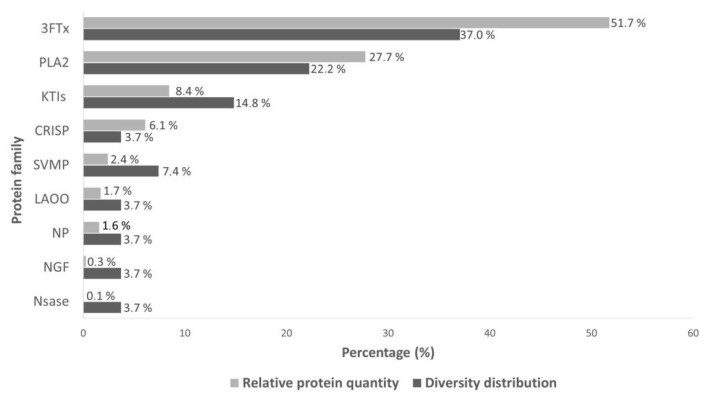
Relative quantification and diversity (isoforms) of the protein classes identified in the proteome of *A. antarcticus* venom. Relative quantification was performed according to the methodology described by [45], and the detailed calculation is provided in Supplementary Material S2 (Table S3). Legend: 3FTxs (three-finger toxins), PLA_2_ (phospholipase A_2_), NP (natriuretic peptide), KTI (Kunitz-type inhibitors), NGF (nerve growth factor), CRISP (cysteine-rich secretory protein), SVMP (metalloproteinase), NSase (nucleotidase), and LAAO (L-amino acid oxidase).

Snake venoms from the Elapidae family are primarily characterized by a high abundance of toxins from the 3FTxs and PLA_2_ families [38], which are recognized for their structural and functional diversity [39,40,41,42,43,44]. In this analysis, this pattern was also observed in the venom of *A. antarcticus*, in which 3FTxs toxins accounted for 51.7% of the proteins identified, followed by PLA_2_, which represented 27.7% of the total (Figure 3). Furthermore, these families also stood out for their diversity, with 37% corresponding to distinct 3FTxs variants (*n* = 10) and 22.2% to different PLA_2_ variants (*n* = 6).

During the analyses, multiple isoforms of these toxins were observed, complicating their precise identification. In some cases, the same toxin was detected in different chromatographic peaks, especially between 10 and 30 min. This pattern may be associated with chromatographic drag or the presence of distinct isoforms of the same protein. Similar findings were described by Birrell (2007), who identified a wide range of unique peptides associated with different PLA_2_ isoforms, highlighting the complexity of these toxins [33]. The presence of multiple isoforms may reflect evolutionary adaptations or functional modularity, thereby broadening the venom’s range of biological targets.

##### Three-Finger Toxins (3FTxs)

Three-finger toxins (3FTxs) are composed of 60 to 74 amino acid residues and exhibit a conserved three-dimensional structure consisting of three loops extending from a central core stabilized by four to five disulfide bridges [46,47]. Variations in the length and conformation of these loops directly influence their interaction with molecular targets, resulting in a broad range of biological functions [48].

3FTxs are classified into three main groups: short-chain (60–62 residues, four disulfide bridges, and a β-hairpin structure), long-chain (66–75 residues, five disulfide bridges, and a helical structure in loop II, typically associated with binding to nicotinic acetylcholine receptors), and non-conventional forms, which exhibit greater variability in size and disulfide bonding patterns [48]. These toxins are associated with neurotoxic, cardiotoxic, anti-platelet aggregation [49], and analgesic effects [50].

Although 3FTxs-type toxins play an important role in the toxicity of snakes from the Elapidae family, their characterization in *A. antarcticus* remains limited. The first 3FTxs from this species was described by Sheumack et al. (1979) as a short-chain neurotoxin capable of affecting muscle action potentials in mice [19]. Subsequently, Kim and Tamiya (1981) identified a long-chain neurotoxin, Alpha-elapitoxin-Aa2b [21], followed in the following year by another short-chain toxin, Short neurotoxin 1 [22], both of which were detected in the present study. Sheumack et al. (1990) described Alpha-elapitoxin-Aa2d, which causes blockade of the postsynaptic neuromuscular junction [20]. Tyler et al. (1997) described Alpha-elapitoxin-Aa2e, a long-chain neurotoxin composed of 79 amino acids [23]. More recently, Blacklow et al. (2011) characterized α-Elapitoxin-Aa2a, which binds to muscle-type nicotinic receptors and was also identified in this work [31]. Since then, no new studies have reported novel 3FTxs in *A. antarcticus*.

In addition, we identified sequences with similarity to transcripts previously reported in *A. wellsi* [34], including “3FTxs-Aca-57”, “3FTxs-Aca-30”, “3FTxs-Aca-27”, “3FTxs-Aca-108”, and “3FTxs-Aca-53”. A sequence with similarity to Alpha-elapitoxin-Nss2a, a toxin described in *Notechis scutatus scutatus*, another Australian elapid from the *Acanthophiinae* subfamily, was also detected.

##### Phospholipase A_2_ (PLA_2_)

PLA_2_s are important components of elapid snake venoms and are widely recognized for their diverse pharmacological activities [41]. Among their most documented biological actions in Elapidae are presynaptic neurotoxicity [51], postsynaptic neurotoxicity [52], cardiotoxicity [53], inhibition of platelet aggregation [54], and induction of edema [55].

These enzymes act by hydrolyzing phosphoglycerides, releasing lysophospholipids and fatty acids [41]. Specifically in Australian elapids, most PLA_2_s are basic, composed of approximately 118 amino acids, with seven disulfide bonds and an average molecular weight of 13 kDa [56].

Compared to 3FTxss, PLA_2_s from *A. antarcticus* were characterized later. The first PLA_2_s described were named “Acanthins” by Van Der Weyden et al. (1997), who reported similarity with neurotoxic PLA_2_s from *Pseudechis porphyriacus* [24]. Later, Chow et al. (1998) further characterized the Acanthin I and II isoforms, which exhibit inhibitory effects on platelet aggregation, both of which were detected in the present analysis [35]. Hains et al. (1999) also contributed by modeling the structure of one of these isoforms to understand its molecular interactions [36]. Blacklow et al. (2010) identified a heterodimeric PLA_2_, termed P-elapitoxin-Aa1a (the sequence of this phospholipase was only partially obtained, and a complete sequencing of the protein was not performed) [30]. Additionally, Birrell (2007), using proteomic analysis after 2D-PAGE, identified PLA_2_s with similarity to those from the genera *Austrelaps*, *Oxyuranus*, *Pseudonaja, Pseudechis*, and *Bitis*, as well as Acanthin I and II [33].

In the present study, we identified similar sequences to Acanthin I and II previously described for *A. antarcticus* [35], as well as transcripts reported by Jackson et al. (2013) in the *A. wellsi* transcriptome [34], including “PLA2-Aca-2”, “PLA2-Aca-34”, and “PLA2-Aca-38”. PLA2-Aca-2 exhibited 44% (peak 2) and 66% (peak 3) sequence similarity with the corresponding *A. wellsi* transcript. PLA2-Aca-38 showed 51% similarity, while PLA2-Aca-34 displayed 34% (peak 15) and 59% (peak 18), respectively. These findings reinforce the presence of PLA_2_ isoforms in the venom of *A. antarcticus* and support the relationship between proteomic and transcriptomic data for species of the *Acanthophis* genus.

#### 2.1.2. Less Abundant Toxins

Toxins from the NP, NGF, SVMP, KTI, CRISP, NSase, and LAAO families are minor components in Elapidae snake venoms [38]. However, despite their low abundance, these molecules may play relevant biological roles. SVMPs, for instance, have been associated with the inhibition of platelets [57]; LAAOs with antibacterial, antiparasitic, and mild anti-platelet effects [58]; NGFs with the modulation of inflammatory processes that enhance the diffusion of other venom toxins [59]; and NSases with the release of adenosine, potentially leading to immunosuppressive and vasodilatory effects [60]. KTIs modulate the hemostatic system and may have neurotoxic activity [61]; NPs act as vasodilators and hypotensive agents [62,63]; and CRISP proteins are known to block ion channels [64]

In the present study, KTIs were the third most abundant class (8.4%), followed by SVMPs (7.4%) and CRISP (6.1%). In contrast, NP (1.6%), NGF (0.3%), NSase (0.1%), and LAAO (1.7%) showed the lowest proportions among the identified proteins. Noteworthy toxins included KP-Aca-8 and KP-Aca-1 (KTIs), as well as SVMP-Aca-4, all of which showed similarity to transcripts previously reported in *A. wellsi*, highlighting the conservation of these toxins among species within the same genus.

Sequences with similarity to Kunitz-type proteins from other Australian snakes, such as *Austrelaps superbus* and *Drysdalia coronoides*, as well as an SVMP similar to Textilase-1, transcribed by *Pseudonaja textilis*. Additionally, sequences similar to NGF from *Notechis scutatus scutatus*, a CRISP from *Drysdalia coronoides*, an NSase from *Naja atra*, and an LAAO previously described in *Pseudechis australis* were identified.

It is noteworthy that although Tan and Ponnudurai (1990) reported NSase activity in *A. antarcticus* venom [65], this toxin family had not yet been identified in previous proteomic studies for the species [32,33]. However, the present study describes for the first time the presence of sequences showing similarity to NSases,, revealing a toxin class not previously detected through proteomic approaches in *A. antarcticus.*

### 2.2. Functional Activity of A. antarcticus Venom

The first reports on the functional activity of *A. antarcticus* venom date back to Calmette (1896), who demonstrated its lethality in guinea pigs and rabbits [14]. Subsequently, Kellaway (1929) described neurotoxic, hemorrhagic, and hemolytic effects and weak anticoagulant activity, in addition to observing, in venom fractions, procoagulant actions and stimulatory effects on smooth muscle [15]. In clinical studies, cases of severe muscle paralysis, ptosis, rhabdomyolysis, lymphadenopathy, and mild edema at the bite site were reported, with no detectable coagulation disorders [16,17]. However, in vitro assays have confirmed a mild anticoagulant activity [15,17,66]

**Figure 4:**
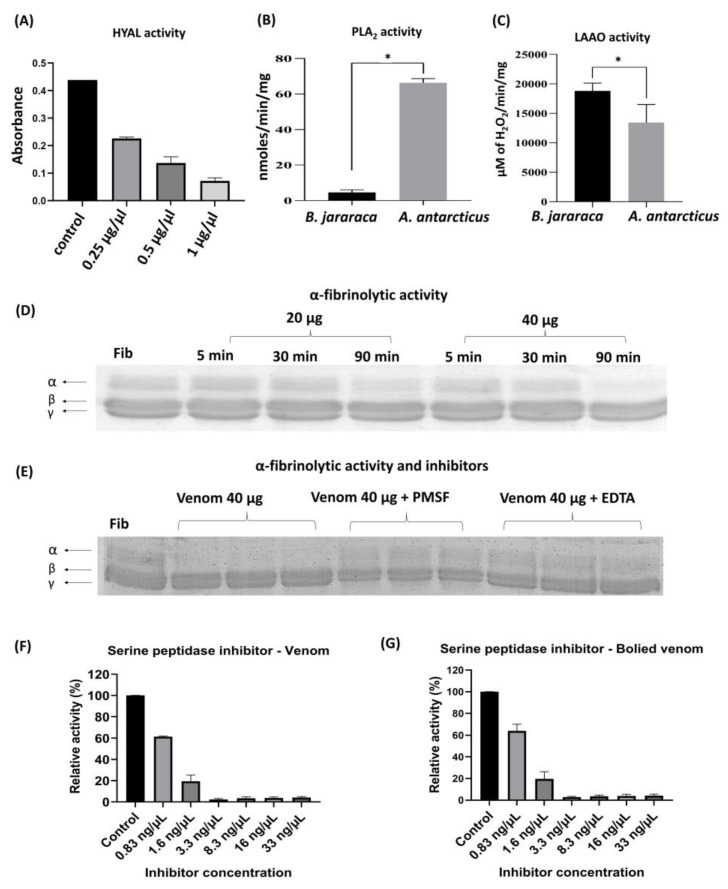
Panel (**A**) demonstrates hyaluronidase (HYAL) activity, evidenced by the hydrolysis of hyaluronic acid at different venom concentrations, with isolated hyaluronic acid used as a negative control; experiments were conducted in duplicate, and the results are expressed as mean ± standard deviation. Panels (**B**,**C**) show the activities of phospholipase A_2_ (PLA_2_) and L-amino acid oxidase (LAAO), respectively, with statistically significant differences (*p* < 0.05), using Bothrops jararaca venom as a positive control. All assays were performed in triplicate, and the results are expressed as mean ± standard deviation. The asterisk (*) indicates statistically significant differences compared to the control group (*p* < 0.05). Panel (**D**) shows the fibrinogenolytic activity of the venom, assessed by SDS-PAGE, where the control sample (Fib) displays intact α, β, and γ fibrinogen chains, while the subsequent lanes represent the effects of venom incubation at different concentrations (20 and 40 µg) and reaction times (5, 30, and 90 min). Panel (**E**) presents the fibrinogenolytic activity of venom incubated with fibrinogen in the presence and absence of the inhibitors PMSF and EDTA, using 40 µg of venom for 90 min, an optimized condition previously established in panel (**D**). As a negative control, isolated fibrinogen (Fib) is shown with preserved α, β, and γ chains. All samples were analyzed in triplicate by SDS-PAGE. Finally, panels (**F**,**G**) illustrate the inhibitory activity against serine peptidase (trypsin) in samples treated with native and heat-denatured venom, respectively, at different concentrations. These assays were performed in duplicate, and the results are expressed as mean ± standard error.

Functional analyses performed with *A. antarcticus* venom revealed activities associated with hyaluronidase (HYAL), PLA_2_, and LAAO and for the first time indicated fibrinogenolytic activity and the presence of serine peptidase inhibitors (Figure 4). To investigate the enzymatic origin of the fibrinolytic activity, specific inhibitors PMSF and EDTA were used, targeting serine peptidases and metallopeptidases, respectively. Significant inhibition was observed under both conditions, suggesting the joint participation of these enzyme classes, an unprecedented finding among Australian snakes. On the other hand, no collagenolytic or caseinolytic activity was detected in the zymography assays (Supplementary Material S3, Figures S3 and S4).

The Hyal, PLA_2_, and LAAO activities observed in this study (Figure 4A–C) are consistent with previous reports. Tan and Ponnudurai (1990) had already described significant activity of these enzymes in *A. antarcticus* venom, along with acetylcholinesterase and weak NSase activity [65]. Tasoulis et al. (2020) also reported these activities, although noting that PLA_2_ is relatively weak compared to other Australian snakes [67].

Regarding proteolytic activity, *A. antarcticus* venom has a low abundance of proteases, as previously described in the literature [65,68]. This feature was confirmed in the present study, with the identification of SVMPs only, which accounted for 2.4% of the total protein content (Figure 3). However, this is the first study to demonstrate fibrinogenolytic activity in this species. The activity was observed after 90 min of incubation, with 20 and 40 µg of venom, being more evident at the higher concentration, and was characterized by selective degradation of the α-chain of fibrinogen (Figure 4D). Based on these results, inhibition assays were conducted exclusively with 40 µg of venom and 90 min of incubation, the conditions under which the activity was most evident.

The data also indicate that the fibrinolytic activity is associated with the presence of SVMPs and SVSPs, based on the results of inhibition assays with PMSF and EDTA (Figure 4E). Although the presence of these proteases has already been described in Australian elapids, generally in low abundance [38], this is the first study to demonstrate their functional activity. SVMPs were identified in the proteomic data, while SVSPs were not detected, possibly due to their very low levels or being below the detection limit of the approach used. Still, the results indicate that, despite being present in low abundance, these peptidases are functionally active and may play secondary roles in the envenomation process.

Lalloo et al. (1996) also investigated the effect of *A. antarcticus* venom on fibrinogen but did not observe its degradation [17]. In contrast, our data revealed α-chain cleavage, which may be attributed to methodological differences between studies. While Lalloo et al. used only 15 min of incubation and lower venom concentrations, this study evaluated longer incubation times (5, 30, and 90 min), with activity detected only after 90 min.

Additionally, inhibitory activity against serine peptidase (trypsin) was observed in both native and heat-treated venom samples, at all tested concentrations (Figure 4F,G). The preservation of this activity even after boiling, a process that promotes thermal denaturation of proteases, suggests that trypsin inhibition is not related to proteolytic activity but rather to the presence of thermostable serine peptidase inhibitors. This activity may be associated with the presence of Kunitz-type inhibitors (KTIs) identified in the proteomic analyses.

### 2.3. Proteomics and Antivenomics

Fry et al. (2002) demonstrated, through mass spectrometry, significant differences in venom composition among species of the genus *Acanthophis* and subspecies of *A. antarcticus* [32]. In a previous study, the same group observed that a monovalent antivenom showed variations in the effectiveness of neutralizing the neurotoxic effects among these different species. However, higher doses ensured complete neutralization of the venoms tested [27]. These interspecific and intraspecific variations are supported by genetic analyses [69] and reinforced by Herzig et al. (2013), who described marked differences among *A. antarcticus* subspecies [70]. Despite the biochemical diversity, antivenom has proven effective against different species of the genus, as long as it is administered in adequate doses [27]. 

In addition, cross-immunity between different species of Australian *Elapidae* has been reported. O’Leary and Isbister (2009) demonstrated that antivenoms labeled for *A. antarcticus* neutralized venoms from other species [71], while Murphy et al. (2024) observed that the tiger snake antivenom neutralized the anticoagulant activity of various venoms [66].

Cross-reactivity among elapid venoms from different continents is largely attributed to the presence of conserved toxins, such as phospholipase A_2_ (PLA_2_), which are considered key contributors to these observed effects [72]. In contrast, three-finger toxins (3FTxs) do not appear to exhibit the same behavior. This hypothesis is supported by Ramos et al. (2017), who showed that an Australian polyvalent antivenom effectively neutralized venoms from eight *Micrurus* species from the Americas in a murine model [73].

These findings emphasize the importance of understanding venom composition and function to identify key toxins responsible for physiopathological effects. Venomic and functional approaches have contributed significantly to the development of more effective and specific antivenoms by focusing on predominant antigens [74,75]. In this context, the application of proteomic techniques, as employed in the present study, is crucial for mapping conserved toxins and their respective activities. This contributes to a deeper understanding of similarities and differences among Elapidae snakes from different continents, thereby clarifying the basis of cross-reactivity observed with Australian antivenoms and supporting the rational design and improvement of future antivenom formulations.

## 3. Conclusions

Our findings represent one of the most comprehensive proteomic analyses ever conducted for the venom of *A. antarcticus*, revealing a complex and diverse molecular composition. The identification of major toxins, such as 3FTxs and PLA_2_, corroborates the expected profile for snakes of the Elapidae family, as well as the presence of less abundant toxins, including NP, KTI, NGF, CRISP, SVMP, NSase, and LAAO. Furthermore, the functional data revealed HYAL, PLA_2_, and LAAO activity. For the first time, inhibitory activity on serine peptidases and fibrinogenolytic activity in the venom of this species were described. The results also demonstrate a similarity between the transcriptome of *A. wellsi* and the proteomic data of *A. antarcticus* presented in this work, suggesting toxin conservation between these species of the genus *Acanthophis*. Additionally, analysis of the hydrophilic fraction of the venom, separated using an HILIC column, allowed the identification of different molecular masses, highlighting the presence of polar components that are usually not detected by reverse phase, the classical approach employed in venomic studies. These findings reinforce the need to expand multiomic studies in poorly characterized Elapidae, aiming to assist in the rational development of antivenoms.

## 4. Materials and Methods

### 4.1. Snake Venom Attainment

The venom of the *Acanthophis antarcticus* snake was acquired from Venom Supplies PTY LTD, located in the Barossa Valley, South Australia. The extraction process involved initially stimulating the snake to bite a parafilm membrane, allowing the venom to flow into a collection tube. A pipette tip was subsequently attached to the snake’s fang for the final extraction phase, ensuring complete collection of the venom. After extraction, the venom was lyophilized to remove moisture, and its dry weight was carefully measured [76]. The samples were then stored at a controlled temperature of −20 °C, preserving their integrity for future experimental use.

### 4.2. Fractionation of Crude Venom by Liquid Chromatography

#### 4.2.1. Reverse-Phase Liquid Chromatography (RP-HPLC)

In total, 5 mg (5 injections) of crude venom was analyzed through liquid chromatography using the Prominence^®^ UFLC Shimadzu system (Kyoto, Japan). The setup included a CBM-20A controller, Sil-20AC HT autoinjector, CTO-20A column oven, LC-20AD pump system, and SPD-20A ultraviolet detector. The analysis utilized a Phenomenex Jupiter C18 column (150 mm × 4.6 mm, 300 Å, 3 µm), with a binary gradient of mobile phase composed of solvent A (water with 0.1% trifluoroacetic acid) and solvent B (90% acetonitrile with 0.1% trifluoroacetic acid). Oven temperature conditions were set at 40 °C with an injection volume of 35 µL (each with 800 µg). Chromatographic separation was carried out over 115 min and monitored at a wavelength of 214 nm. The method included the following steps: 0–5 min with 5% B, 5–20 min with 25% B, 20–80 min with 45% B, 80–90 min with 70% B, maintaining this condition until 95 min, followed by reconditioning from 95–105 min with 5% B and maintaining until 115 min. Six collections from each peak were performed. The samples obtained were lyophilized and subjected to in-solution digestion.

#### 4.2.2. Hydrophilic Interaction Liquid Chromatography (HILIC-HPLC)

A 5 mg crude venom was subjected to a solid-phase extraction process (SPE) using a C18 phase-filled cartridge (100 mg/mL) from Applied Separations (Allentown, PA, USA). Two solvents were used for the elution: (A) water containing 0.1% trifluoroacetic acid and (B) 90% acetonitrile and 0.1% acetic trifluoracetic. The fraction not retained during the SPE was carefully collected and later lyophilized. After this process, the final sample was resuspended in acidified water and quantified using the BCA protocol (Thermo Scientific^TM^, São Paulo, Brazil).

From this final sample, 500 µg was separated by high-efficiency liquid chromatography at hydrophilic phase (HILIC-HPLC) using a Kinetex column (250 mm × 4.6 mm × 100 Å, 5 µm). The binary system of the mobile phase was composed of solvent A (water containing 5 mM of ammonium format) and solvent B (95% acetonitrile with 5 mm of ammonium format). The column temperature was adjusted to 50 °C and the injection volume to 50 µL. The chromatographic analysis consisted of a gradient of 0–3 min with 100% B, 3–20 min with 70% B, keeping this condition up to 23 min, and then reconditioning 23–27 min with 100% B. All the analysis was monitored with a wavelength of 214 nm and later analyzed by MALDI-TOF.

### 4.3. Fingerprint Analysis of HILIC-HPLC Samples by Mass Spectrometry

MALDI-TOF mass spectrometry was employed to obtain a molecular fingerprint of the peptides and proteins present in the collected fractions from HILIC-HPLC. The analysis was performed using the AXIMA Performance system from Shimadzu^®^, operating in linear mode and using a universal matrix. The mass range was set between 300 and 50.000 *m*/*z*. Instrument parameters were configured as follows: laser power at 80, 200 profiles per acquisition, and 5 shots per measurement. In total, 1 ug per sample was used for this analysis.

### 4.4. Proteomic Analysis of RP-HPLC Samples by Mass Spectrometry

#### 4.4.1. Sample Preparation: In-Solution Digestion

The sample was prepared for proteomic analysis through reduction and alkylation protocol [77]. It was resuspended in 50 µL of ammonium bicarbonate buffer (NH_4_HCO_3_, 100 mM) pH 8 and treated with 3 µL of dithiothreitol (DTT, 100 mM), followed by incubation at 30 °C for 1 h in a dry bath. Next, 4 µL of iodoacetamide (IAA, 200 mM) was added and incubated at room temperature in darkness for 45 min. To finalize the process, 1 µL of trypsin (1 µg/µL) was introduced, and the reaction proceeded at 37 °C for 16 h. The reaction was then halted by the addition of 5 µL of trifluoroacetic acid.

#### 4.4.2. Mass Spectrometry Analysis

Proteomic analysis was conducted using the LCMS-IT-TOF Shimadzu system, equipped with a Phenomenex Kinetex C18 column (50 mm × 4.6 mm, 2.6 µm). A binary mobile phase system was utilized, consisting of solvent A (water with 0.1% acetic acid) and solvent B (90% acetonitrile with 0.1% acetic acid). The chromatographic conditions were set at an oven temperature of 40 °C and an injection volume of 45 µL. The method program was as follows: 0–5 min at 0% B, 5–80 min at 40% B, maintained until 84 min, 84–130 min at 100% B, maintained until 140 min, followed by reconditioning from 140 to 150 min at 0% B, which was maintained until the conclusion at 160 min. Mass spectrometry conditions employed an electrospray ionization in positive mode with 4.5 Kv in the ion source at 200 °C, and MS/MS experiments were performed with CID-type fragmentation set at 70%. Precursor ion acquisition was performed within the *m*/*z* range of 330–1900, while the fragment ion range was set to 350–1400 *m*/*z*.

#### 4.4.3. Data Processing and Analysis

The proteomic data were analyzed using PEAKS Studio V7.0 software (Bioinformatics Solutions Inc., Waterloo, ON, Canada). Carbamidomethylation of cysteine was designated as a fixed modification, methionine oxidation was treated as a variable modification, and trypsin was specified as the cleavage enzyme. Fragment ion tolerance was set at 0.1 Da. Up to three post-translational modifications per peptide were permitted, and a stringent false discovery rate (FDR) threshold of less than 0.5% was applied. The data analysis was conducted using a comprehensive dataset, comprising revised Uniprot entries together with those associated with the *Acanthophiinae* taxon. InterPro V102.0 was used to investigate the molecular and biological functions of each identified protein.

### 4.5. Biological Assays

All biological tests were conducted with crude venom and previously quantified by BCA protocol (Thermo Scientific^TM^).

#### 4.5.1. Hyaluronidase Activity

Hyaluronidase activity was evaluated according to a protocol adapted from Pukrittayakamee et al. (1988) [78]. The assay was performed in a 96-well plate previously cooled on ice for 10 min. Each well received 40 µL of hyaluronic acid solution (0.5 mg/mL) prepared in sodium acetate buffer (0.2 M sodium acetate with 0.15 M NaCl, pH 6.0). Different venom concentrations (0.25, 0.5, and 1 µg/µL) were used, and 10 µL of each was added to the respective wells. As a negative control, hyaluronic acid without venom was used. The plate was incubated at 37 °C for 15 min, and the reaction was stopped by adding 150 µL of CTAB solution (2.5% in 2% NaOH) to each well. Samples were analyzed in duplicate, and the results were expressed as mean ± RSD.

#### 4.5.2. Phospholipase A_2_ Activity

Phospholipase A2 (PLA2) activity was evaluated based on the methodology described by Holzer and Mackessy (1996) [79]. The test was performed in a 96-well plate, in which 200 µL of Tris-HCl buffer (pH 8.0) containing 10 mM of CaCl_2_ and 100 mM NaCl was deposited, along with 40 µL of the sample pre-prepared by the dissolution of 20 µg of venom in saline at 0.85% *m*/*v*. Then, 20 µL of the 4-nitro-3-octanoloxi-benzoic acid (4-NOBA) synthetic substrate was incorporated to reach a final concentration of 0.32 mM (using a 4.16 mM stock solution on acetonitrile). The plate was incubated at 37 °C for 60 min, and the absorbance reading was performed at 425 nm. For the calculation of the specific activity, it was considered that an absorbance of 0.01 corresponds to the release of 25.8 nmoles of chromophore release and that a PL_A2_ activity unit is equivalent to 1 nM of chromophore released. Thus, the specific activity was expressed in chromophore/min/mg of venom. The sample was analyzed in triplicate, and the results were expressed as average ± RSD.

#### 4.5.3. L-Amino Acid Oxidase Activity

The enzymatic activity of L-amino oxidase (LAAO) was assessed following the methodology described by Kishimoto and Takahashi [80]. For this assay, 10 µL of venom (at a concentration of 1 mg/mL) was mixed with 90 µL of reaction buffer, composed of 50 mM Tris-HCl (pH 8.0), 250 mM L-methionine, 2 mM o-phenylenediamine, and 0.8 U/mL horseradish peroxidase. The reaction was incubated at 37 °C for 60 min. After the incubation period, the reaction was terminated by adding 50 µL of 2M H_2_SO_4_. Absorbance measurements were recorded at a wavelength of 492 nm. LAAO activity was indirectly determined using a standard curve of H_2_O_2_, with the results expressed in µM of H_2_O_2_ produced/min/mg of venom. The sample was analyzed in triplicate, and the results were expressed as average ± RSD.

#### 4.5.4. Fibrinogenolytic Activity

The fibrinogenolytic activity [81] assay was performed by incubating 20 40 µg and 40 µg of venom with 200 µL of human fibrinogen (2 mg/mL) at 37 °C. Aliquots were collected at 5, 30, and 90 min of reaction. The samples were then prepared in a reduction buffer containing 4% *m*/*v* SDS, 20% *v*/*v* glycerol, and 20% *v*/*v* mercaptoethanol. Subsequently, they were analyzed using SDS-PAGE electrophoresis with a 10% polyacrylamide gel [82]. For the inhibitor analyses, samples containing 40 µg of venom incubated for 90 min in the presence of 20 mM PMSF or 10 mM EDTA were used.

#### 4.5.5. Serine Peptidase Inhibitor

The inhibition assay for serine peptidases was conducted using a 96-well plate containing 100 ng of trypsin (14 nM), 150 μL of the fluorogenic substrate Z-Phe-Arg-AMC (0.1 mM), and samples of boiled and non-boiled venom at different concentrations (33; 16; 8.3; 3.3; 1.6; and 0.86 ng/μL). The reaction mixture also included 100 mM Tris-HCl buffer (pH 8.0) with 20 mM CaCl_2_, bringing the final reaction volume to 300 μL. Two controls were included: (1) a reaction containing trypsin, substrate, and buffer and (2) one with trypsin, substrate, buffer, and 10 mM benzamidine, a known serine peptidase inhibitor. After incubation at 37 °C for 30 min, fluorescence measurements were performed using a spectrofluorometer, with excitation and emission wavelengths set at 330 nm and 430 nm, respectively, and an auto-cutoff at 420 nm. The sample was analyzed in duplicate, and the results were expressed as average ± SEM.

## Figures and Tables

**Table 1:** Identification of chromatographic peaks of *A. antarcticus*.

Peaks	Protein Class	Description	Taxon Homology	Accession
1	Three-finger toxins	Short neurotoxin 1	*Acanthophis antarcticus*	P01434
2	Three-finger toxins	3FTxs-Aca-57	*Acanthophis wellsi*	R4G2D8
		3FTxs-Aca-55	*Acanthophis wellsi*	R4FI70
		Alpha-elapitoxin-Aa2a	*Acanthophis antarcticus*	P86522
		Short neurotoxin 1	*Acanthophis antarcticus*	P01434
	Phospholipase A_2_	PLA_2_ -Aca-2	*Acanthophis wellsi*	R4FI60
	Natriuretic peptide	NP-Aca-7	*Acanthophis wellsi*	R4G7E4
3	Phospholipase A_2_	PLA_2_ -Aca-2	*Acanthophis wellsi*	R4FI60
	Three-finger toxins	Alpha-elapitoxin-Aa2a (Fragment)	*Acanthophis antarcticus*	P86522
		3FTxs-Aca-57	*Acanthophis wellsi*	R4G2D8
4	Three-finger toxins	3FTxs-Aca-57	*Acanthophis wellsi*	R4G2D8
		3FTxs-Aca-27	*Acanthophis wellsi*	R4G2D9
	Phospholipase A_2_	Basic phospholipase A_2_ acanthin-2	*Acanthophis antarcticus*	P81237
		Basic phospholipase A_2_ acanthin-1	*Acanthophis antarcticus*	P81236
		Basic phospholipase A_2_ PA-13	*Acanthophis wellsi*	P04057
5	Three-finger toxins	Alpha-elapitoxin-Aa2b	*Acanthophis antarcticus*	P01385
		Alpha-elapitoxin-Nss2a	*Notechis scutatus scutatus*	P01384
		3FTxs-Aca-30	*Acanthophis wellsi*	R4G7E8
		3FTxs-Aca-57	*Acanthophis wellsi*	R4G2D8
6	Three-finger toxins	3FTxs-Aca-30	*Acanthophis wellsi*	R4G7E8
	Kunitz inhibitor	Kunitz-type serine protease inhibitor 18	*Drysdalia coronoides*	F8J2F6
7	Three-finger toxins	3FTxs-Aca-27	*Acanthophis wellsi*	R4G2D9
		3FTxs-Aca-108	*Acanthophis wellsi*	R4G2P2
8	Three-finger toxins	Alpha-elapitoxin-Aa2b	*Acanthophis antarcticus*	P01385
		3FTxs-Aca-27	*Acanthophis wellsi*	R4G2D9
9	Three-finger toxins	3FTxs-Aca-108	*Acanthophis wellsi*	R4G2P2
		Alpha-elapitoxin-Aa2b	*Acanthophis antarcticus*	P01385
	Kunitz inhibitor	KP-Aca-8	*Acanthophis wellsi*	R4G2D6
		Kunitz-type serine protease inhibitor superbin-2	*Austrelaps superbus*	B5KL39
10	Three-finger toxins	Alpha-elapitoxin-Aa2b	*Acanthophis antarcticus*	P01385
	Kunitz inhibitor	KP-Aca-1	*Acanthophis wellsi*	R4FI64
	Phospholipase A_2_	Basic phospholipase A_2_ acanthin-2	*Acanthophis antarcticus*	P81237
11	Phospholipase A_2_	Basic phospholipase A_2_ acanthin-2	*Acanthophis antarcticus*	P81237
	Three-finger toxins	3FTxs-Aca-53	*Acanthophis wellsi*	R4FI75
12	Phospholipase A_2_	PLA_2_ -Aca-38	*Acanthophis wellsi*	R4G7E3
13	Phospholipase A_2_	Basic phospholipase A_2_ acanthin-2	*Acanthophis antarcticus*	P81237
14/15	Venom nerve growth factor	Venom nerve growth factor 3	*Notechis scutatus scutatus*	Q3HXY5
	Phospholipase A_2_	PLA_2_ -Aca-34	*Acanthophis wellsi*	R4G2D4
16	Phospholipase A_2_	PLA_2_ -Aca-34	*Acanthophis wellsi*	R4G2D4
17/18	Cysteine-rich protein	Cysteine-rich venom protein	*Drysdalia coronoides*	F8J2D4
19/20	Metallopeptidase	SVMP-Aca-4	*Acanthophis wellsi*	R4G2D3
	Nucleotidase	Snake venom 5′-nucleotidase (Fragment)	*Naja atra*	A0A2I4HXH5
21	L-amino acid oxidase	L-amino acid oxidase	*Pseudechis australis*	Q4JHE1
	Metallopeptidase	SVMP-Aca-4	*Acanthophis wellsi*	R4G2D3
22	L-amino acid oxidase	L-amino acid oxidase	*Pseudechis australis*	Q4JHE1
	Metallopeptidase	SVMP-Aca-4	*Acanthophis wellsi*	R4G2D3
23	L-amino acid oxidase	L-amino acid oxidase	*Pseudechis australis*	Q4JHE1
	Metallopeptidase	Textilease-1	*Pseudonaja textilis*	B5KFV9

## Data Availability

The original contributions presented in this study are included in the article/supplementary material. Further inquiries can be directed to the corresponding author.

## Supplementary Materials

The following supporting information can be downloaded at: https://www.mdpi.com/article/10.3390/toxins17080405/s1, Supplementary Materials S1—Figure S1: Percentage of proteins reviewed in the UniProt database in the different subfamilies of the Elapidae family, Figure S2: Percentage of proteins reviewed in the UniProt database in the different genera within the subfamily Acantophiinae; Supplementary Materials S2—Table S1: Proteins, Table S2: Fragments, Table S3: Quantification; Supplementary Materials S3—Figure S3: Zymography, casein activity, Figure S4: Zymography, gelatin activity.
