# Supplementary material for: Functional and Proteomic Characterization of Acanthophis antarcticus Venom: Evidence of Fibrinogenolytic and Serine Peptidase Inhibitory Activities
Source: Toxins (Basel). 2025 Aug 13;17(8):405. doi: 10.3390/toxins17080405 (PMC12389826; doi:10.3390/toxins17080405)
Supplement: Supplementary file 1 [file toxins-17-00405-s001.zip › Supplementary Materials S1.pdf]

Figure S1 - Percentage of proteins reviewed in the UniProt database in the different subfamilies of the *Elapidae* family.

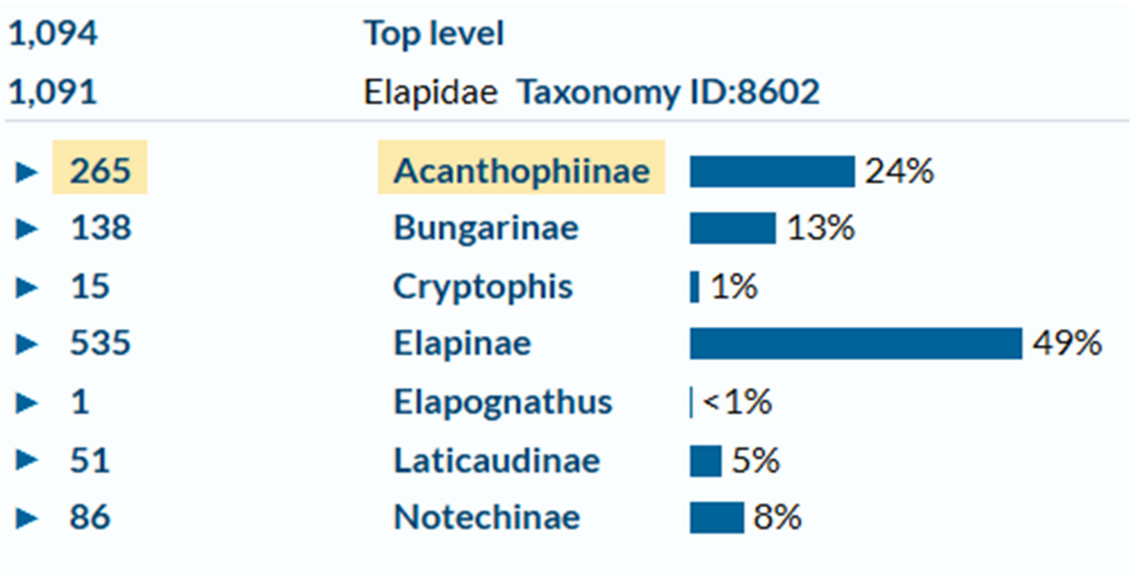

https://www.uniprot.org/uniprotkb?facets=reviewed%3Atrue&groupBy=taxonomy&parent=8602&query=elapidae (accessed on 31 March 2025).

Figure S2 - Percentage of proteins reviewed in the UniProt database in the different genera within the subfamily *Acantophiinae*

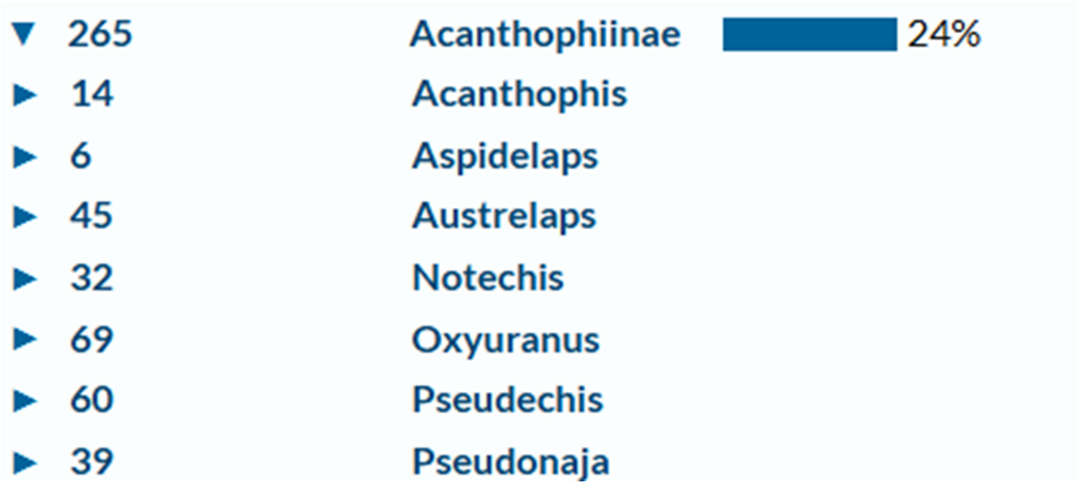

https://www.uniprot.org/uniprotkb?facets=reviewed%3Atrue&groupBy=taxonomy&parent=8602&query=elapidae (accessed on 31 March 2025).
