# Supplementary material for: Functional and Proteomic Characterization of Acanthophis antarcticus Venom: Evidence of Fibrinogenolytic and Serine Peptidase Inhibitory Activities
Source: Toxins (Basel). 2025 Aug 13;17(8):405. doi: 10.3390/toxins17080405 (PMC12389826; doi:10.3390/toxins17080405)
Supplement: Supplementary file 1 [file toxins-17-00405-s001.zip › Supplementary Materials S3.pdf]

Functional and Proteomic Characterization of *Acanthophis Antarcticus* Venom: Evidence of Fibrinogenolytic and Serine Peptidase Inhibitory Activities

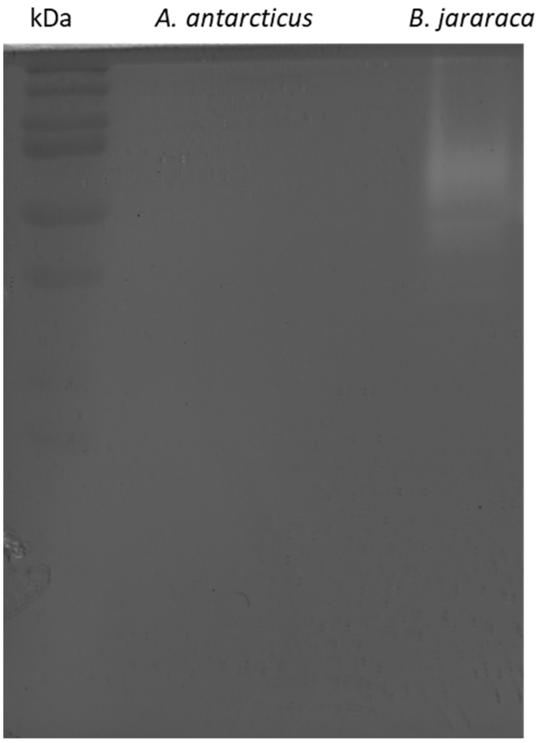

Figure S3 – Zymography, casein activity

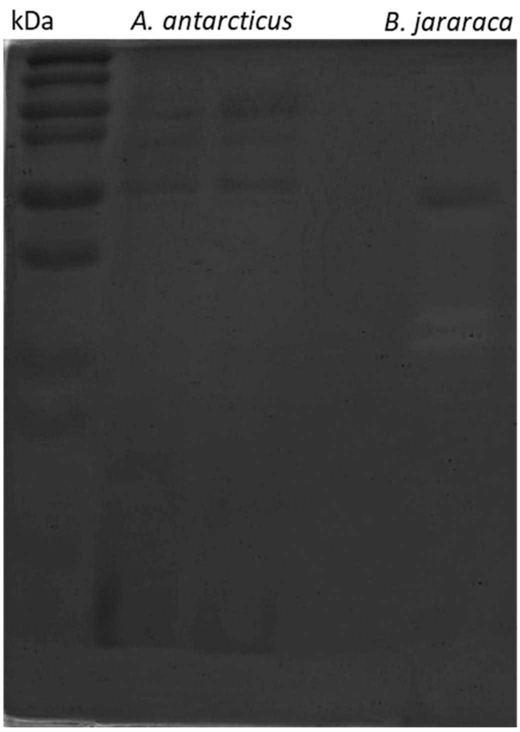

Figure S4 – Zymography, gelatin activity

Proteolytic activity of the venom was assessed by zymography using 10% polyacrylamide gels containing 2 mg/mL of gelatin or casein as substrate. For each assay, 40 µg of venom were diluted in non-reducing sample buffer (62.5 mM Tris-HCl, pH 6.8; 2% SDS; 10% glycerol; 0.01% bromophenol blue), without prior heating. Samples were loaded onto gels and subjected to conventional SDS-PAGE under non-reducing conditions at 120 V. After electrophoresis, the gels were incubated for 1 h at room temperature in renaturation buffer containing 2.5% Triton X-100, followed by two washes with distilled water for 10 minutes each. Subsequently, the gels were incubated for 18 h at 37 °C in incubation buffer (50 mM Tris-HCl, 140 mM NaCl, 5 mM $CaCl_2$; pH 7.4). Proteolytic activity was visualized by staining the gels with Coomassie Brilliant Blue R-250.
